# Protection of Cattle against Rinderpest by Vaccination with Wild-Type but Not Attenuated Strains of Peste des Petits Ruminants Virus

**DOI:** 10.1128/JVI.00040-16

**Published:** 2016-04-29

**Authors:** Barbara Holzer, Sophia Hodgson, Nicola Logan, Brian Willett, Michael D. Baron

**Affiliations:** a The Pirbright Institute, Pirbright, Surrey, United Kingdom; b MRC-University of Glasgow Centre for Virus Research, Glasgow, Scotland, United Kingdom

## Abstract

Although rinderpest virus (RPV) has been eradicated in the wild, efforts are still continuing to restrict the extent to which live virus is distributed in facilities around the world and to prepare for any reappearance of the disease, whether through deliberate or accidental release. In an effort to find an alternative vaccine which could be used in place of the traditional live attenuated RPV strains, we have determined whether cattle can be protected from rinderpest by inoculation with vaccine strains of the related morbillivirus, peste des petits ruminants virus (PPRV). Cattle were vaccinated with wild-type PPRV or either of two established PPRV vaccine strains, Nigeria/75/1 or Sungri/96. All animals developed antibody and T cell immune responses to the inoculated PPRV. However, only the animals given wild-type PPRV were protected from RPV challenge. Animals given PPRV/Sungri/96 were only partially protected, and animals given PPRV/Nigeria/75/1 showed no protection against RPV challenge. While sera from animals vaccinated with the vaccine strain of RPV showed cross-neutralizing ability against PPRV, none of the sera from animals vaccinated with any strain of PPRV was able to neutralize RPV although sera from animals inoculated with wild-type PPRV were able to neutralize RPV-pseudotyped vesicular stomatitis virus.

**IMPORTANCE** Rinderpest virus has been eradicated, and it is only the second virus for which this is so. Significant efforts are still required to ensure preparedness for a possible escape of RPV from a laboratory or its deliberate release. Since RPV vaccine protects sheep and goats from PPRV, it is important to determine if the reverse is true as this would provide a non-RPV vaccine for dealing with suspected RPV outbreaks. This is probably the last *in vivo* study with live RPV that will be approved.

## INTRODUCTION

The year 2011 saw the final declaration of the global eradication of rinderpest, one of the most devastating cattle diseases the world has known. This was the first livestock disease, and only the second viral disease, ever eradicated, and the global benefits of rinderpest eradication are estimated to be in the billions of dollars (1). That is not to say that rinderpest virus (RPV) itself has disappeared from the world. A number of laboratories are known to have isolates of wild-type RPV for scientific or historic reasons or due to lapses in freezer stock control. Accidental release of RPV from such a laboratory is thought to be the most likely pathway by which the virus might reenter the environment (2, 3) although it might also be deliberately released as an act of sabotage or bioterrorism, whether as a natural isolate or as one made by synthetic biology based on the RPV genome sequences already in databases.

One of the problems with these rinderpest vaccines is that there is no way of telling, serologically, which animals have recovered from disease and which have been vaccinated; in other words, there is no accepted vaccine able to distinguish infected from vaccinated animals (termed a DIVA vaccine). In an emergency response, it may be necessary to kill all vaccinated animals in order to be sure that all traces of RPV have been eliminated. The impact of a reemergence would be lessened if a good DIVA vaccine existed which could be deployed. A policy of rapid barrier “vaccination to live,” which spares protected animals, could then be implemented as soon as there was a genuine likelihood that RPV had reemerged. In the absence of a DIVA vaccine, use of the existing RPV vaccine is likely to be delayed until there has been reference laboratory confirmation of RPV, and implementation could be further delayed/inhibited if it is known that even vaccinated animals will later be culled to reinstate the RPV-free status.

RPV is a member of a small group of viruses of the genus Morbillivirus, which includes measles virus (MV), canine distemper virus (CDV), and peste des petits ruminants virus (PPRV). PPRV causes a severe disease in sheep and goats, and it was found early on that giving the rinderpest vaccine to sheep and goats protected them from PPRV (4). Subsequently, attenuated forms of PPRV were developed to use as vaccines (5, 6), and these are now used globally to control PPR disease. One such vaccine was also shown to elicit an immune response in sheep and goats that would prevent the replication of RPV in those animals (7). What has not been tested is whether the PPRV vaccine strains could protect cattle against RPV. If one or more of the PPRV vaccines protect cattle against rinderpest, they would act as effective DIVA vaccines. Well-established tests exist that can distinguish between anti-RPV and anti-PPRV antibodies (8–10). So it would be possible to distinguish clearly between RPV-infected and PPRV-vaccinated animals, and it would still be possible to screen for RPV infections during and after vaccination. In addition, the natural reluctance to deploy rinderpest vaccine, even in the case of a suspected reappearance/release of rinderpest, would not be a problem if we could deploy one of the widely used PPRV vaccines instead.

Another advantage of being able to use PPRV vaccines against RPV would be that we would no longer have to maintain stocks of rinderpest vaccine around the world, and these could be eliminated, reducing costs for at-risk countries as well as eliminating a potential source of contamination for laboratory samples. Total removal of RPV from as many facilities as possible is the best solution to this problem. PPRV vaccines are in production in a number of countries, and it would be possible to produce and put into the field large amounts of such vaccine at very short notice in the case of even a suspected rinderpest reappearance.

Because of these potential benefits, a test of the ability of PPRV vaccines to protect cattle from rinderpest disease was therefore approved by the World Organisation of Animal Health (OIE) and the United Nations Food and Agriculture Organization (FAO), despite the general prohibition on carrying out *in vitro* or *in vivo* work with live RPV. We show here that while wild-type PPRV can protect cattle from challenge with wild-type RPV, neither of the two most widely used vaccine strains of PPRV was able to protect vaccinated cattle, even when used at 20 doses of vaccine per animal.

## MATERIALS AND METHODS

### Cells and viruses.

Vero-derived cells lines were as previously described (11). Wild-type PPRV/Ivory Coast/89 (IC89) and PPRV/Nigeria/75/1 (N75) were grown on Vero cells expressing canine SLAM (Vero-dog-SLAM, or VDS, cells); a cloned RPV vaccine strain (the Plowright vaccine, also sometimes referred to as RBOK) (RPvacc) (12) was grown on Vero cells expressing human SLAM (Vero-human-SLAM, or VHS, cells); PPRV/Sungri/96 (S96) was a gift from MSD Animal Health; RPV challenge virus (RPV/Saudi/81) (RPwt, where wt is wild type) was a previously validated stock of lyophilized spleen from an infected cow. All viruses were titrated on VDS cells (in general, we observed a 20-fold increase in titer of wild-type PPRV/RPV stocks determined on VDS cells compared to results on unmodified Vero cells).

### Animal studies.

All animal studies were carried out under licenses issued by the Home Office of the United Kingdom in accordance with relevant legislation and after approval by The Pirbright Institute (TPI) Animal Welfare and Ethical Review Board.

For study 1, two groups of goats (9 to 12 months old), each consisting of five animals, were inoculated with a standard dose (2 × 10^4^ 50% tissue culture infectious doses [TCID_50_] determined in VDS cells [VDS TCID_50_], equivalent to 10^3^ TCID_50_ determined in Vero cells) of S96 or cloned N75, given subcutaneously. Two cohoused animals were left unvaccinated to act as controls for the challenge virus. After 4 weeks, the animals were infected with 2 × 10^5^ VDS TCID_50_ of IC89, also given subcutaneously. Blood samples were collected on various days postvaccination (dpv) and postchallenge infection (dpi) for preparation of serum.

For study 2, 30 male Holstein-Friesian cattle (approximately 4 months old) were used in five groups of 6 animals each. Four groups were vaccinated by subcutaneous injection with different viruses: (i) 2 × 10^4^ VDS TCID_50_ of RPvacc, (ii) 4 × 10^5^ VDS TCID_50_ of IC89, (ii) 4 × 10^5^ VDS TCID_50_ of S96, and (iv) 4 × 10^5^ VDS TCID_50_ of N75. One group of animals was kept as unvaccinated controls. Blood samples were collected on various dpv and dpi for serum, for the preparation of peripheral blood mononuclear cells (PBMCs), and for viral RNA extraction. PBMCs were prepared by density gradient centrifugation on Ficoll-Paque (GE Healthcare Life Sciences) using standard protocols and frozen in aliquots for later assay of T cell proliferation.

Clinical scores for both PPR and RP disease were calculated based on rectal temperatures and other clinical signs. A score of 1 was given for rectal temperatures 0.5 to 1.9°C above normal for the animal, and a score of 2 was given for temperatures >1.9°C above normal. Similarly, a score of 1 was given for ocular or nasal congestion, congestion of the gums, a soft stool, or apathetic behavior; a score of 2 was given for visible ocular/nasal discharge, 1 to 2 lesions in the gums, fluid diarrhea, or failure to eat; a score of 3 was given for profuse ocular/nasal discharge, necrotic oral lesions, blood in the diarrhea, or failure to stand. The cumulative score on each day was recorded as the final clinical score.

### Antibody neutralization of RPV and PPRV.

All sera were heated at 56°C for 2 h before assay to inactivate any remaining virus in the serum as well as to inactivate complement. Virus-neutralizing titers of sera from infected animals were assayed in 96-well plates by standard procedures (13). The target viruses were N75 for PPRV-neutralizing antibodies and RPvacc for RPV-neutralizing antibodies, and the cells were VDS cells. The neutralizing titer was expressed as the reciprocal of the antibody dilution at which 50% of the wells showed virus growth.

### Antibody quantitation by cELISA.

A competition enzyme-linked immunosorbent assay (cELISA; The Pirbright Institute) for the PPRV hemagglutinin protein (PPRV-H) and a PPRV nucleoprotein (PPRV-N) cELISA (IDVet) were performed according to the manufacturers' protocols.

### VSV pseudotype-based assay for virus neutralizing antibody.

Recombinant vesicular stomatitis virus (VSV) in which the glycoprotein (G) gene has been deleted (VSVΔG) and replaced with firefly luciferase (*luc*) has been described previously (14, 15) and was kindly provided by Michael Whitt, Memphis, TN. An initial stock of VSVΔG *luc* bearing VSV-G was used to infect 293T cells transfected with the VSV-G expression vector pMDG (16). VSVΔG *luc* (VSV-G) pseudotypes were recovered, titrated on 293T cells, and used to prepare a working stock of VSVΔG *luc* (VSV-G) pseudotypes. To prepare constructs expressing PPRV-H and the PPRV fusion protein (PPRV-F), the complete H and F open reading frames were amplified from the N75-encoding plasmid pCI-PPRV-delL (17) using the primers PPRV-H-NotWtF (5′-CCGGCGGCCGCACCATGTCCGCACAAAG-3′) and PPRV-H-BamH1R (5′-GGGGGATCCTCAGACTGGATTACATGTT-3′) for the H gene and PPRV-F-Wt-NotF (5′-GGGGCGGCCGCACCATGCATGCGCCGA-3′) and PPRV-F-BamH1-WtR (5′-GGGGGATCCGCCTACAGTGATCTCACGT-3′) for the F gene. The rinderpest H cDNA was amplified in two steps from the Kabete O strain plasmid pT7KOH. First, the primers RPHsalMUTFwd (5′-GGACGTCGACATGACCATGATTACGCCAAGCTCTAATACGACTCACTATAGGGAAAGCTTGCATGCCTGCAGA-3′) and RPHsalmutREV (5′-TAAGCGTCTACCCTGTCTCTTG-3′) were used to amplify a short product that eliminated an SalI cloning site. The product of this amplification was then used in conjunction with the primer RinderpestHnotrev (5′-GCATGCGGCCGCCTATTTCCCATTGCAAG-3′) to amplify the full-length H cDNA. The rinderpest F cDNA was amplified from pT7 KOF using the primers RinderpestFsalFwd (5′-GCAAGTCGACATGAAGATCTTATTTGC-3′) and RinderpestFnotrev (5′-GCATGCGGCCGCCTACAGTGACCGTACGTA-3′). All amplifications used an Expand High Fidelity PCR system (Roche) and were performed under the following thermocycling conditions: denaturation at 94°C for 5 min, followed by 35 cycles of 94°C for 30 s, annealing at 50°C to 64°C (depending on the midpoint temperature [*T_m_*] of the primers) for 60 s, and extension at 72°C for 120 s, with a final extension at 72°C for 10 min. Products were digested with the enzymes BamHI and NotI and cloned into the eukaryotic expression vector VR1012 (Vical, Inc.). The nucleic acid sequences of all amplified cDNAs were determined externally by Sanger dideoxy chain termination sequencing (LIGHTrun Sequencing Service, GATC Biotech AG, Cologne, Germany). All oligonucleotide primers were obtained from Integrated DNA Technologies, Leuven, Belgium.

To prepare VSVΔG pseudotypes incorporating a luciferase marker gene and bearing the envelope glycoproteins of either PPRV or RPV [VSVΔG *luc* (PPRV) or VSVΔG *luc* (RPV), respectively], 293T cells were transfected with either the PPRV-H and -F expression constructs or those for RPV-H and -F, followed by superinfection with VSVΔG *luc* (VSV-G) as described previously (14, 15). Supernatants were harvested at 48 h postinfection, aliquoted, and frozen at −80°C. The titer of each viral pseudotype stock was estimated by preparing serial dilutions in triplicate and plating the stock onto 293dogSLAM cells, followed by incubation for 48 to 72 h at 37°C, at which time luciferase substrate was added (Steadylite Plus; PerkinElmer), and the signal was analyzed on a Microbeta 1450 Jet luminometer (PerkinElmer). The TCID_50_ of the pseudotyped virus was calculated using the Spearman-Kärber formula (18). Inhibition of luciferase expression from the VSVΔG *luc* pseudotypes by experimental serum was determined as previously described (19), and the neutralization titer was defined as the reciprocal of the serum dilution at which the luciferase yield was reduced by 50%.

### T cell proliferation assay.

Proliferation of T cells in response to virus antigen was measured by a modification of the method of Lund et al. (20). Virus-specific antigen was prepared from VDS cells infected with N75 or RPvacc at a multiplicity of infection (MOI) of 0.01 and incubated until syncytia were well established. The infected cells were scraped into their medium and centrifuged at 2,500 rpm for 20 min. The pellet was resuspended in sterile phosphate-buffered saline (PBS), and virus was inactivated at 56°C for 2 h. Virus antigen was extracted by three rounds of sonication for 1 min and centrifugation for 20 min at 7,600 rpm in a microcentrifuge, pooling the supernatants at each extraction. The pooled supernatant was then spun at 28,000 rpm for 2.5 h in a Beckman 70.1 Ti rotor, and the final pellet was resuspended in PBS–10% sucrose. Protein concentration was determined using Bradford reagent (Bio-Rad). Uninfected VDS cells were processed in the same way to create the control antigen.

PBMCs from vaccinated cattle were cultured in RPMI medium containing 10% fetal calf serum (FCS), 1× nonessential amino acids, 1 mM sodium pyruvate, 50 μM 2-mercaptoethanol, and 10 μg/ml gentamicin. Cells were distributed in round-bottomed 96-well plates at 2 × 10^5^ cells per well and cultured for 6 days in the presence of 20 μg/ml specific or control antigen. [^3^H]thymidine (37 kBq) was added to each well 18 h before the end of the incubation. Cells were harvested onto filters, and the incorporated ^3^H was measured by scintillation counting. A stimulation index was calculated as the ratio of counts incorporated in cells incubated with specific antigen to counts incorporated in cells incubated with the control antigen.

### RT-qPCR for RPV RNA.

Whole blood was taken in EDTA Vacutainers. Total RNA was extracted using an LSI MagVet Universal Isolation kit (Thermo Fisher) on a MagMax Express-96 processor. RPV RNA was assayed by quantitative reverse transcription-PCR (RT-qPCR) in 5 μl of the extracted RNA using AgPath-ID one-step RT-PCR reagents and a modification of the primer/probe set L10 described by Carrillo and colleagues (21), in which the probe had a 3′ quencher/minor groove binder (Thermo Fisher) in place of the 3′ 6-carboxytetramethylrhodamine (TAMRA) quencher used in the original study, as this was required to allow the probe to work at 60°C. Results are expressed as the mean 40-*C_T_* value, where *C_T_* is the threshold cycle. Each blood sample was extracted twice, and each extract was assayed by RT-qPCR in duplicate.

### Statistical analysis.

Statistical analysis of experimental data was carried out using standard functions in R. In most cases, data were fit to a general linear or polynomial model using the function lme, with individual animals as random factors, and multiple pairwise comparisons were carried out using the Tukey analysis option of the glht function. T cell proliferation data did not fit the normality expectation and so were analyzed by a Kruskal-Wallis nonparametric test with correction for multiple comparisons (function kruskalmc).

### Rinderpest biocontainment.

All studies with live RPV and PPRV were carried out in the containment facilities of The Pirbright Institute. The laboratories and animal units operate a biosecurity standard specific for group 4 (high-impact) livestock pathogens that are not harmful to humans, as described in the document Animal Pathogens: Guidance on Controls” issued by the United Kingdom Department for Environment, Food, and Rural Affairs (22). In addition, the facilities are designed to meet or exceed the “Minimum Standards for Laboratories working with FMD *in vitro*/*in vivo*,” a standard adopted by the 38th General Session of the European Commission for the Control of Foot-and-Mouth Disease on 30 April 2009 (23). The Pirbright Institute is an approved FAO-OIE rinderpest holding facility, and the study with live rinderpest virus was approved by the FAO and OIE.

## RESULTS

### Validation of PPRV vaccine strains.

**FIG 1:**
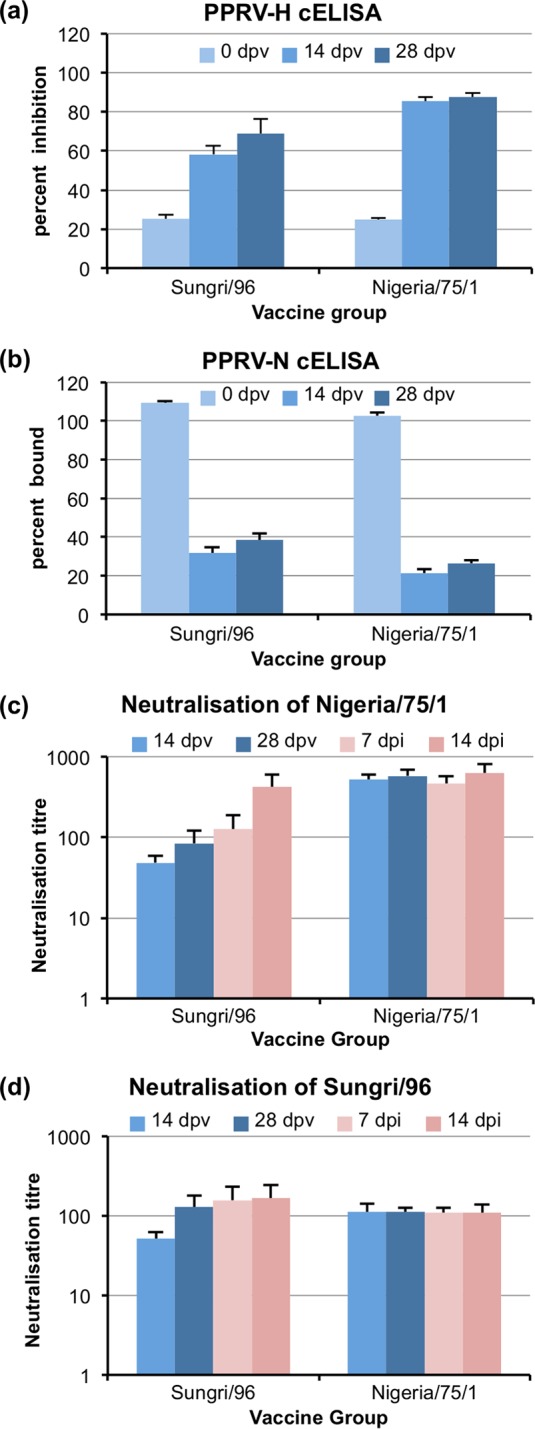
Antibody response in goats to PPRV vaccine stocks. Goats were vaccinated with either the Sungri/96 or Nigeria/75/1 vaccine strain of PPRV as described in Materials and Methods. Sera collected at the indicated days postvaccination (dpv) or postinfection (dpi) with wild-type PPRV were assayed for anti-PPRV antibodies by cELISA using the PPRV-H protein-specific ELISA kit (a), cELISA using the PPRV-N protein cELISA kit (b), neutralization of the infection of VDS cells by PPRV Nigeria/75/1 (c), and neutralization of the infection of VDS cells by PPRV Sungri/96 (d). Blue bars indicate samples taken before challenge with wild-type PPRV; pink bars indicate samples taken after challenge. Error bars show 1 standard error. (Note that due to differences in the way that the two cELISA kits calculate their results, a positive result in the PPRV-H cELISA is indicated by an increase in the plotted value [percent inhibition], while a positive result in the PPRV-N cELISA is indicated by a decrease in the plotted value [percent bound]).

The PPRV vaccine strains chosen for the study were the original vaccine strain derived from isolate PPRV/Nigeria/75/1 (N75) (5), used throughout Africa, the Middle East, and most of Asia, and the most commonly used of those derived in India, Sungri/96 (S96) (24). In order to be sure that our vaccine stocks were functional, they were first tested in goats to show that they were normally immunogenic and gave protection against wild-type PPRV. Five goats were vaccinated with each PPRV vaccine strain. Antibody responses to vaccination were checked by competition ELISA (cELISA) (Fig. 1a and b) and also by virus neutralization titer (VNT) (Fig. 1c and d). In the latter case, VNTs were determined separately against both N75 and S96. All vaccinated animals gave strong anti-PPRV antibody responses. The antibodies raised by the two vaccines neutralized either virus. Although the sera from animals vaccinated with N75 neutralized N75 more effectively (higher functional titer) than any other combination (Fig. 1c and d), there was no significant difference between the results for any other combination of vaccine and neutralizing target. The antibodies elicited by N75 were better at displacing monoclonal antibody from ELISA antigen prepared from N75 virus (Fig. 1a) or bacterially expressed antigen based on the N75 sequence (Fig. 1b). However, it was clear that both vaccine preparations were immunogenic, and there was strong cross-reaction between the PPRV vaccines from different lineages.

**FIG 2:**
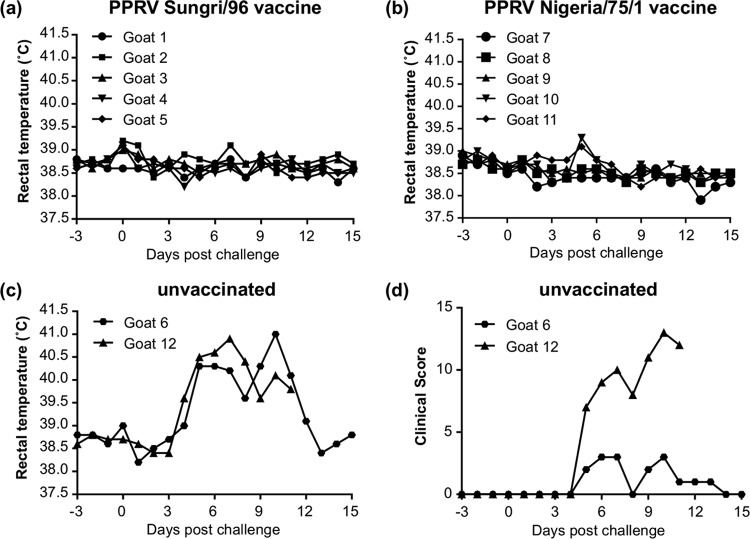
Protection of goats from virulent PPRV challenge by PPRV vaccines. Goats vaccinated with PPRV as described in the legend of Fig. 1 were challenged with virulent PPRV/Ivory Coast/89 at 28 days after vaccination. (a and b) Rectal temperatures of the two groups of vaccinated animals. (c) Rectal temperatures of the two unvaccinated control animals. (d) Clinical scores of the two unvaccinated control animals. Animal 12 was euthanized at 11 days postchallenge. Clinical scores of vaccinated animals were zero at all time points.

After 4 weeks, all the goats, plus two unvaccinated control animals, were challenged with IC89, which has previously been found to be pathogenic in goats in the United Kingdom (25, 26). Both unvaccinated animals developed full PPR disease, and one had to be euthanized at 11 days postchallenge (Fig. 2). None of the vaccinated animals showed any clinical signs (Fig. 2), indicating that the vaccine stocks were working as expected.

### Serum response to PPRV strains in cattle.

To assess immune responses in cattle to PPRV, groups of six Holstein-Friesian cattle (4 to 5 months old) were vaccinated with (i) the standard Plowright RPV vaccine strain (27) (RPvacc), (ii) 20× the normal dose of N75, (iii) 20× the normal dose of S96, or (iv) the same dose (4 × 10^5^ TCID_50_) of IC89 or were left unvaccinated. The immune response to the inoculated viruses was observed over 4 weeks, after which the animals were challenged with wild-type RPV (RPwt); for this we used RPV/Saudi/81, one of the most virulent RPV isolates ever characterized (28) and the challenge virus used in our previous studies with RPV (12, 29–31).

**FIG 3:**
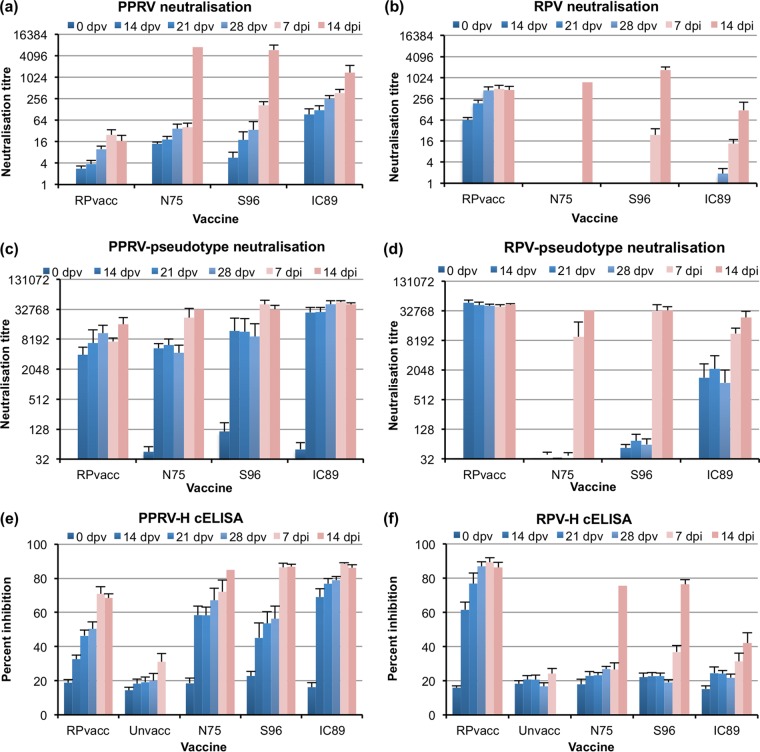
Antibody response in cattle to vaccination with PPRV strains or RPV vaccine. Cattle were inoculated with RPV vaccine or different PPRV strains as described in Materials and Methods. Sera were collected at the indicated days postvaccination (dpv) or postinfection (dpi) with virulent RPV. Serum antibodies recognizing RPV or PPRV were assayed before (blue bars) and after (pink bars) challenge with virulent RPV. Error bars show 1 standard error (no standard error could be calculated for the N75 group at 14 dpi since only one animal survived to that point). (a) Neutralization of the infection of VDS cells by PPRV Nigeria/75/1. (b) Neutralization of the infection of VDS cells by RPV vaccine. (c) Neutralization of PPRV-pseudotyped VSV entry into 293-SLAM cells. (d) Neutralization of RPV-pseudotyped VSV entry into 293-SLAM cells. (e) cELISA using a PPRV-H protein-specific ELISA kit. (f) cELISA using an RPV-H protein-specific ELISA kit. As negative serum has a known nonspecific effect in the cELISAs, we show also the results for the unvaccinated animals for these assays.

Sera taken from experimental animals were tested for their ability to inhibit cell infection by RPV (RPvacc) and PPRV (N75) in standard microneutralization assays (Fig. 3). All animals inoculated with one of the strains of PPRV developed PPRV-neutralizing antibodies (Fig. 3a). Interestingly, the antibodies elicited by the RPV vaccine also neutralized PPRV. The titer of neutralizing antibody against N75 appeared highest in animals given IC89, which had statistically significantly higher titers than those seen in animals given S96 or RPvacc (*P* < 0.001). The titer observed in animals inoculated with N75 was intermediate, not significantly different from those elicited by either S96 or IC89 though significantly higher than the titers in animals given RPvacc (*P* = 0.017).

When the sera were tested against RPV, a very different picture emerged. While the RPV vaccine strain, as expected, elicited a good titer of RPV-neutralizing antibodies, none of the antibodies elicited in any of the experimental animals by any of the PPRV strains had RPV-neutralizing activity (Fig. 3b), with the exception of sera from 2 of the 6 animals given IC89, which showed very low RPV-neutralizing titers (<10) at 28 dpv. In all other PPRV sera, RPV-neutralizing titers were below detectable limits.

In case there were neutralizing antibodies which were present at levels below those detectable by the traditional microneutralization assay, we also assayed neutralizing antibodies using a more sensitive vesicular stomatitis virus (VSV) pseudotype system (19) based upon a replication-defective VSV (VSVΔG) incorporating a luciferase marker gene and bearing the envelope glycoproteins of either PPRV or RPV. Using such pseudotyped viruses in which the envelope glycoproteins were those of PPRV (N75 strain) and RPV (the Kabete O strain from which RPvacc was derived), we found a similar picture (Fig. 3c and d) as for the classic neutralization assay but with increased sensitivity at low titers of specific antibodies. Perhaps because of this higher sensitivity, the assay also saturated at very high titers (>1/32,000). All the vaccinated animals had anti-PPRV activity by this assay although there was no statistical difference between the N75, S96, and RPvacc groups. The IC89 group again had higher anti-PPRV activity than any of the others (*P* < 0.01). The higher sensitivity of this assay at low titers of specific antibody enabled us to clearly show the presence of RPV-neutralizing antibodies in the sera of animals vaccinated with IC89. The levels of anti-RPV antibody were lower in the IC89 group than in the RPV group (*P* < 0.001) but higher than in either the S96 (*P* = 0.002) or N75 (*P* < 0.001) group. Although there was a suggestion in the data that the S96 group also had some RPV-neutralizing activity (Fig. 3c and d), results for this group were not statistically different from those for the N75 group. None of the N75-vaccinated animals had significant (titer of >32) anti-RPV activity.

The antibody responses in the vaccinated animals were also examined by a different type of serological assay, measuring the ability of antiserum to compete for specific epitopes on the viral H proteins in a competition ELISA (cELISA). Sera were tested for activity in the H protein-specific cELISAs for anti-RPV and anti-PPRV antibodies (8). The data from these assays correlated well with the data from the neutralization assays. All animals given PPRV developed anti-PPRV-H antibodies (Fig. 3e). In addition, animals immunized with RPvacc developed antibodies which cross-reacted in the PPRV cELISA, as previously reported (8). Anti-PPRV-H activity was highest in the animals given IC89 and was statistically higher than that seen for the group given S96 (*P* = 0.003) or that given RPvacc (*P* < 0.001). The titers in the group given N75 were again intermediate, being significantly higher than those of the RPvacc group (*P* = 0.002) but not significantly different from those of either the S96 group or the IC89 group.

It was previously shown that the RPV-H protein cELISA is entirely specific for RPV-directed antibodies (8), and that was also observed here, with a strong anti-RPV antibody activity in the sera from the RPvacc group; no anti-RPV activity was detected in any of the other groups prior to challenge (Fig. 3f).

### Proliferative (T cell) responses to PPRV strains in cattle.

**FIG 4:**
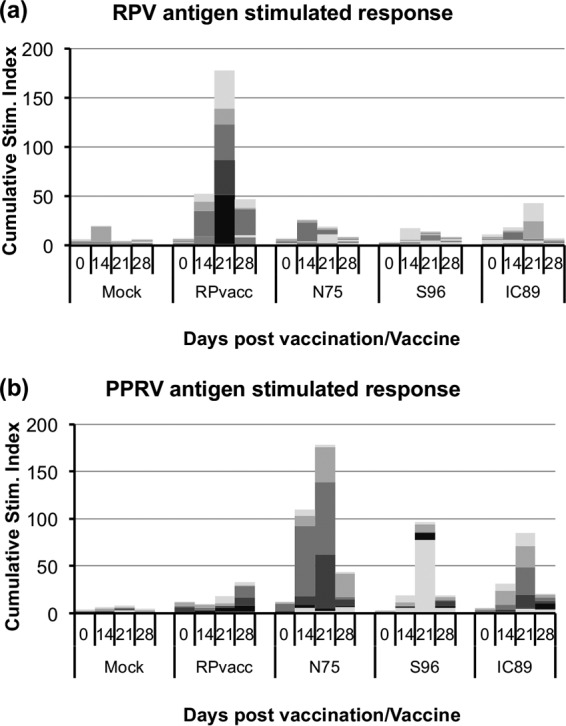
Assay of T cell proliferation in PBMCs from vaccinated animals. PBMCs were isolated from cattle vaccinated as described in the legend of Fig. 3. Proliferation in response to stimulation with RPV or PPRV proteins was measured as described in Materials and Methods and plotted as the cumulative stimulation index (ratio of thymidine incorporation in response to specific antigen to incorporation in response to control antigen) for each group of animals at the indicated day postvaccination. The response in individual animals is shown by different shades of gray, which make up the cumulative response.

The generation of RPV- or PPRV-specific T cells was studied by looking at the stimulation of proliferation of PBMCs incubated with viral antigen (20). Responses in these outbred animals were, as expected, variable, and the proliferative responses to the vaccine viruses were weak; nevertheless, we found that animals vaccinated with RPvacc generated a clear T cell response to RPV antigen (Fig. 4a). Similarly, animals vaccinated with IC89 or N75 showed a proliferative response to PPRV antigen (Fig. 4b). The response in the animals vaccinated with S96 was not statistically significantly different from that in unvaccinated animals. The proliferative response to PPRV antigen appeared slightly stronger in the N75 group, which may reflect the homologous nature of the antigen used to stimulate proliferation. A weak RPV-specific response was seen also in some of the animals vaccinated with different strains of PPRV (Fig. 4a), but this was statistically significant only for the IC89 group.

### Challenge with wild-type RPV.

**FIG 5:**
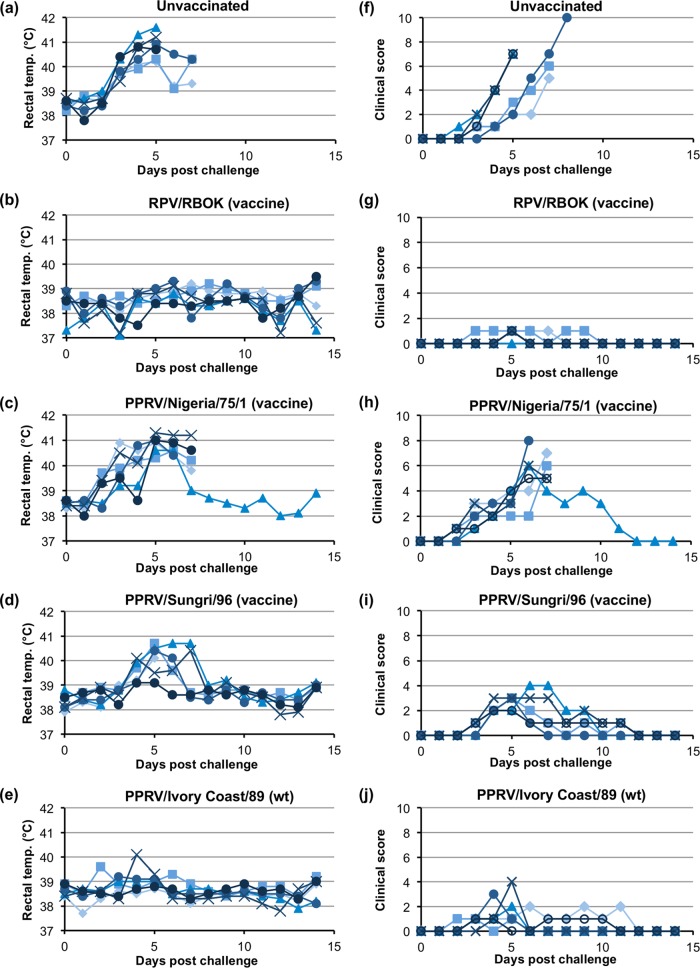
Effect of challenge with virulent RPV on vaccinated animals. Cattle were left unvaccinated (a and f) or were vaccinated with the indicated vaccine and wild-type (wt) viruses (b to e and g to j). All the cattle were infected with virulent RPV at 3 weeks postvaccination. (a to e) The rectal temperatures for individual animals in each group are shown. (f to j) The clinical score on each day was calculated as described in Materials and Methods, and the scores for individual animals in each group are shown.

The vaccinated and unvaccinated animals were challenged by inoculation with RPwt. The unvaccinated controls developed classic rinderpest disease, with high temperatures, ocular and nasal discharge, and in several cases gum lesions and diarrhea (Fig. 5). All of these animals were euthanized by 7 dpi. The animals previously given the RPV vaccine strain were protected from disease, showing no pyrexia and no significant clinical signs (some animals in all groups developed slight congestion in the eyes, which may have been a result of dust from hay feed but was not related to any other sign of rinderpest disease). The group of animals inoculated with IC89 were protected from rinderpest disease, with no significant difference in the temperature profiles or mean clinical scores between this group and the group given the RPV vaccine strain (*P* < 0.001). In contrast, neither of the PPRV vaccine strains was able to protect animals from disease. Animals vaccinated with N75 were almost indistinguishable from the group of unvaccinated animals (Fig. 5), with 5 out of 6 animals having to be euthanized by 7 dpi. The S96 vaccine gave partial protection such that the animals in this group displayed a milder set of clinical signs, less extreme pyrexia, and resolution of the disease by the end of the study. There was no significant difference in the mean clinical scores for the N75 and unvaccinated groups, while there were significant differences between those for the S96 and N75 (*P* < 0.001) groups and between the S96 and unvaccinated (*P* < 0.001) groups. However, all animals in the S96 group showed classic rinderpest disease signs and were clearly significantly sicker (higher mean clinical score) than animals in the IC89 or RPvacc group ((*P* < 0.001).

### Effect of vaccination on viremia in RPV-challenged animals.

**FIG 6:**
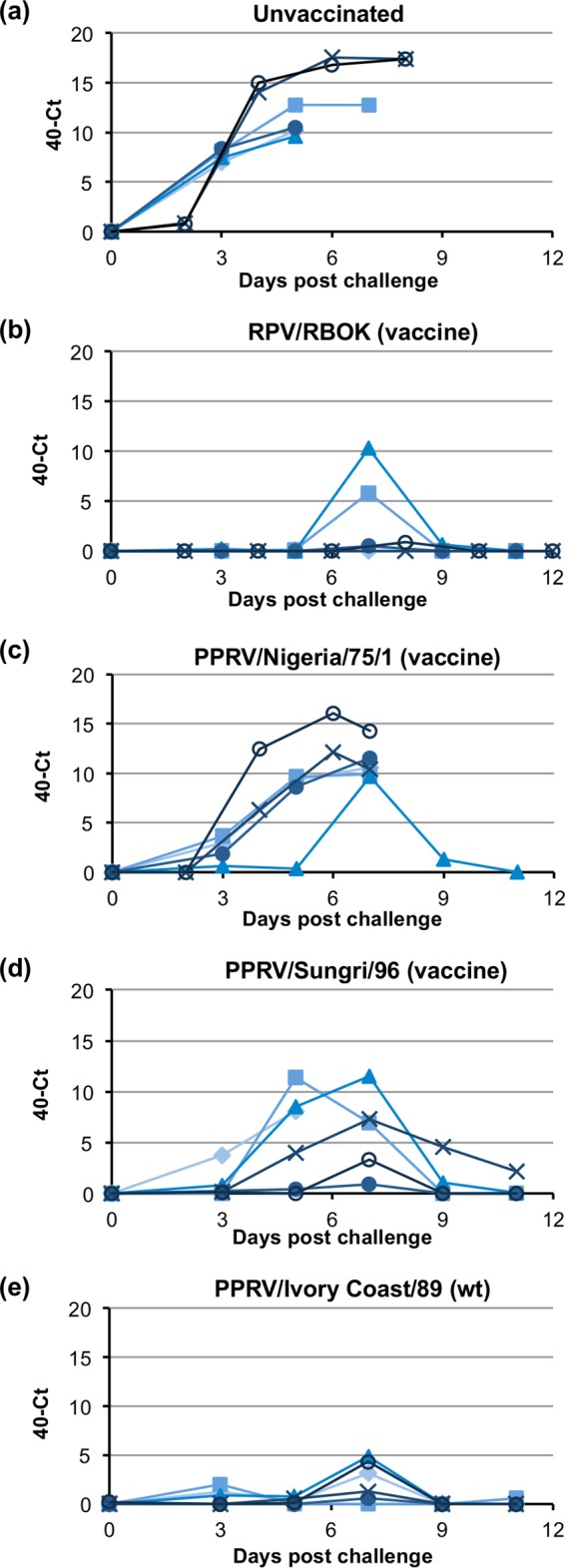
Replication of wild-type RPV in vaccinated cattle. Cattle were left unvaccinated (a) or were vaccinated with the indicated vaccine or wild-type (wt) virus (b to e). All the cattle were infected with virulent RPV at 3 weeks postvaccination. RNA was extracted from whole blood taken from experimental animals at different times after challenge with virulent RPV. RPV-specific RNA was assayed by reverse-transcription/real-time PCR (RT-qPCR), and the amount of viral RNA is expressed as the mean 40-*C_T_* value, a value which increases as the amount of viral RNA increases. Values for individual animals in each group are shown.

The replication of the challenge virus in the experimental animals was further characterized by measuring rinderpest virus RNA in blood samples taken at approximately 2-day intervals over the challenge period. Total RNA was extracted from blood, and the rinderpest virus RNA was measured by one-step reverse-transcription real-time PCR. The threshold cycle (*C_T_*) was taken as a measure of the relative amount of viral RNA in the blood of each animal on the respective days (Fig. 6). In the two groups showing severe disease (unvaccinated and vaccinated with N75), virus RNA climbed sharply at around 3 dpi, peaking at around 6 dpi. Interestingly, even though the clinical state of the animals continued to deteriorate after 6 dpi, the level of virus in the blood did not increase, and in some animals it declined. This may reflect a continued loss of white cells in the blood of animals suffering severe rinderpest disease. The animals that were protected from disease (vaccinated with RPvacc or IC89) showed no RPV RNA or only very low levels, which disappeared by 9 dpi. As was the case with the clinical scores, the animals vaccinated with S96 presented an intermediate picture, with higher and more prolonged viremia in some animals than seen in any in the RPvacc and IC89 groups but significantly lower levels than seen in animals in the unprotected groups. There was no statistical difference between the viremia levels seen in the RPvacc and IC89 groups, while for each of these the level was clearly lower than the level seen in the unvaccinated or N75-vaccinated group (*P* < 0.001 for all). There was a statistically significant difference between results for the unvaccinated and those for the N75-vaccinated groups (*P* = 0.0047), reflecting the fact that one animal in the latter group survived. The viremia in the S96 group was not statistically different from that in the RPvacc or IC89 group but was lower than the level seen in either the N75 group (*P* = 0.024) or the unvaccinated group (*P* < 0.001).

### Antibody responses in RPV-challenged animals.

After challenge, anti-RPV antibodies could be detected in the sera of all of the PPRV-inoculated animals that survived, whether measured as RPV-neutralizing antibody (Fig. 3b) or as neutralization of the RPV pseudotyped VSV (Fig. 3d) or in the RPV-H cELISA (Fig. 3f). Animals that were fully protected (RPvacc or IC89) showed no, or only a small, increase over the level seen before challenge, while the N75- and S96-vaccinated animals showed a stronger boost in RPV-neutralizing antibodies. The cross-neutralizing ability of anti-RPV antibodies on PPRV, seen previously after vaccination (Fig. 3a and c), was again observed, with animals originally inoculated with the PPRV vaccine strains showing an increase in PPRV neutralizing activity (Fig. 3a), as well as slight increases in PPRV pseudotype neutralization. Just as the antibodies elicited by vaccination with RPvacc were positive in the PPRV-H cELISA (Fig. 3e), so the RPV challenge gave rise to antibodies that increased the activity in this assay.

## DISCUSSION

Cross-reaction, cross-neutralization, and cross-protection between different morbilliviruses have been studied for many years and in many different animals, primarily focusing on the well-established relationships between measles virus (MV), canine distemper virus (CDV), and RPV (32–37). Much of our knowledge comes from early studies as the relationships between these viruses were established by such functional studies and before molecular techniques became available. It is well established that naturally occurring or specifically induced antiserum against any morbillivirus cross-reacts with proteins of any other virus in the same genus (33, 35, 38–42). However, cross-protection induced by different morbilliviruses is not always a reciprocal relationship and depends a great deal on the virus strains and animals used, so that Goret et al. found that wild-type CDV could protect cattle from RPV while RPV provided only partial protection of ferrets against CDV (32). On the other hand, DeLay et al. found that RPV protected dogs from CDV, but the wild-type CDV did not protect cattle from RPV (34). While such differences in observed cross-protection may be due to variation in the ability of some strains of virus to replicate sufficiently in a heterologous host to trigger a potent immune response, they may also indicate fundamental differences in the breadth of the immune response in specific cases.

Previous studies on RPV and PPRV have shown that inoculation of goats with the N75 PPRV vaccine could elicit antibodies that neutralized RPV in cell culture, albeit at a lower titer than the neutralization of PPRV (7). On the other hand, treatment of goats with the RPV vaccine strain elicited antibodies that neutralized RPV, as expected, but showed no, or only trace, neutralizing ability against PPRV (4). Similar to these findings, we found that none of the PPRV strains used in our study elicited antibodies that neutralized RPV infection in cell culture. The presence of good titers of PPRV-neutralizing antibodies, as well as antibodies that were positive in the PPRV-specific cELISA, indicated that all three PPRV strains replicated and were immunogenic in the cattle. Despite the low levels of anti-RPV antibodies and the absence of significant amounts of classically RPV-neutralizing antibodies, animals inoculated with IC89 showed essentially complete protection against RPV challenge. This is a similar picture to the findings of Taylor with RPV in goats or to the findings of several authors that a single inoculation of MV in dogs elicited MV-neutralizing but not CDV-neutralizing antibodies (37, 43), while protecting the dogs against CDV (34, 36), or the recent finding that inoculating macaques with CDV or MV elicited antibodies that were not cross-neutralizing (44). In the studies reported here, we have used a more sensitive test for neutralizing antibodies, based on VSV pseudotypes; this assay revealed the presence of significant anti-RPV activity in the IC89-vaccinated animals prior to challenge. Cross-protection may therefore have been mediated by a weak humoral response (not detected by traditional neutralizing assay techniques), coupled with an associated cell-mediated response.

The main aim of the study was to see if either of the PPRV vaccine strains could protect against RPV infection in cattle. Our data show clearly that they did not. Although both vaccine strains replicated in the cattle and elicited PPRV-neutralizing antibodies and PPRV-specific T cells, the best that was achieved was a partial suppression of RPV replication and consequent clinical signs in the group of animals inoculated with S96. Animals inoculated with N75 were essentially indistinguishable, in their levels of clinical rinderpest disease and viremia, from those which were unvaccinated. It will therefore not be possible to replace stocks of RPvacc being held for emergency use with stocks of PPRV vaccine.

An important question is why RPvacc protects goats against PPRV while the reciprocal protection is not observed for the PPRV vaccine strains. The most likely reason is the essentially random nature of the attenuating mutations that arise in these vaccine strains during serial passage in cell culture. Both the S96 and N75 vaccines have been safe and effective in sheep and goats over a number of years of use, suggesting that both have acquired a number of separate attenuating mutations. The presence of multiple attenuating mutations prevents the reversion to virulence of the strains through any single random base change. Since the nature of these attenuating mutations is uncharacterized, there was always the possibility that one or more of them would have a particularly strong effect on the replication or immunogenicity of the virus in cattle. This was the reason that we tested two independently developed PPRV vaccine strains and also included a wild-type, unattenuated strain of PPRV. Our data show that there is sufficient immunological similarity between RPV and PPRV for cross-protection since IC89 protected the cattle from RPV. This finding is in accord with very early studies, from before the virus that causes PPR was isolated and characterized, where crude material from animals suffering from PPR was shown to protect cattle from rinderpest disease (45). However, we found that the vaccine strains of PPRV are partially (S96) or completely (N75) unable to induce this immune protection in cattle. There is precedent for such an effect of attenuation in vaccine strains in that wild-type CDV protected cattle against RPV, but an attenuated vaccine strain of CDV did not offer such protection (32). It remains theoretically possible, therefore, that targeted attenuation of a wild-type PPRV isolate to create a new vaccine strain could create one that cross-protected against RPV in cattle, but such development work would not be justified, given the relatively low risk of RPV outbreaks occurring.

The cross-protection observed between morbilliviruses has been ascribed to cell-mediated immunity rather than to cross-reacting antibody (36, 37). In our studies we were indeed able to demonstrate statistically significant RPV-specific T cell-mediated responses in IC89-inoculated cattle, which was the group that showed protection against RPV challenge. However, the responses were weak, and no statistically significant difference could be shown between results for the different groups of PPRV-inoculated animals. There may have been additional RPV-specific T cells in the lymph nodes but in insufficient numbers in the circulating cell population to give a definite response in our assay. Our use of young animals may have mitigated against detecting the cell-mediated response since similar studies showed no detectable cell-mediated immune response to CDV in young pups given MV, while it was easily detected when older animals were used (37). The data shown in Fig. 4 do suggest a slight RPV-specific response in the PBMCs from PPRV-inoculated animals, and more sensitive tests, such those measuring the proliferation of purified CD4^+^ or CD8^+^ cells extracted from the PBMCs, may reveal a better cell-mediated immune response to PPRV infection in cattle that reacts to RPV antigen and may discriminate better between the different groups.

Similar to other studies on morbillivirus cross-protection, our study did not show a correlation between classically assayed RPV-neutralizing antibody and protection against RPV. None of the PPRV-vaccinated animals had detectable RPV-neutralizing antibodies but showed complete, partial, or no protection against RPV, depending on the PPRV strain used. The protection against RPV that was observed appeared to correlate with the anti-RPV antibody activity detected by the very sensitive VSV pseudotype inhibition assay. It may be that very low levels of cross-reactive B cell selection/activation are all that is required to provide protection against disease and that the anti-RPV activity we have observed explains the protection against disease provided by IC89 and the limited protection provided by S96. Another possibility is that cross-protection is indeed mediated by antibody, but not antibody that neutralizes virus directly. There may be epitopes on the surface of morbilliviruses, particularly on the highly conserved fusion glycoprotein (42, 46), that elicit cross-reactive antibodies that fix complement, lead to killing of infected cells, or otherwise inhibit virus growth while not preventing virus infection in cell culture. It is known that antibodies to the RPV-F protein do not neutralize virus directly but only in the presence of complement (47), while similar anti-RPV-H antibodies are directly neutralizing. Immunofluorescence studies have previously shown the presence of antibodies in measles serum that recognize the surface glycoproteins of CDV in the absence of CDV-neutralizing activity (40).

We have shown that wild-type PPRV protects cattle against RPV challenge and that this group had significant anti-RPV antibodies and RPV-specific T cell responses not detected in the groups given either of two PPRV vaccines. It is known from many recent serum surveys (48–52) that PPRV circulating in the small ruminant population can subclinically infect cattle, giving rise to PPRV-specific antibodies in the sera. It is interesting to speculate whether such subclinical infections occurred in the past, affecting the spread of RPV, and similarly whether any of the extensive spread of PPRV in the last 15 years has been due to the eradication of RPV. The more-or-less constant presence of measles virus (either wild type or vaccine) in human populations for the last 2,000 years may have protected us from disease caused by CDV, which has now shown itself able to cause disease outbreaks in primates (53, 54). Determining the exact mechanism of cross-protection between morbilliviruses will require more extensive characterization of the range of antibodies elicited in infection as well as more detailed dissection of the cell-mediated immune response.
